# An Overview of Molecular Mechanism of Nephrotic Syndrome

**DOI:** 10.1155/2012/937623

**Published:** 2012-07-11

**Authors:** Juliana Reis Machado, Laura Penna Rocha, Precil Diego Miranda de Menezes Neves, Eliângela de Castro Cobô, Marcos Vinícius Silva, Lúcio Roberto Castellano, Rosana Rosa Miranda Corrêa, Marlene Antônia Reis

**Affiliations:** ^1^Pathology Laboratory, Department of Biological Sciences, Federal University of Triângulo Mineiro, 38025-180 Uberaba, MG, Brazil; ^2^Immunology Laboratory, Department of Biological Sciences, Federal University of Triângulo Mineiro, 38025-180 Uberaba, MG, Brazil; ^3^Technical Health School of UFPB, Federal University of Paraíba, 58051-900 João Pessoa, PA, Brazil

## Abstract

Podocytopathies (minimal change disease (MCD) and focal segmental glomerulosclerosis (FSGS)) together with membranous nephropathy are the main causes of nephrotic syndrome. Some changes on the expression of nephrin, podocin, TGF- *β* , and slit diaphragm components as well as transcription factors and transmembrane proteins have been demonstrated in podocytopathies. Considering the pathogenesis of proteinuria, some elucidations have been directed towards the involvement of epithelial-mesenchymal transition. Moreover, the usefulness of some markers such as TGF- *β* 1, nephrin, synaptopodin, dystroglycans, and malondialdehyde have been determined in the differentiation between MCD and FSGS. Experimental models and human samples indicated an essential role of autoantibodies in membranous glomerulonephritis, kidney damage, and proteinuria events. Megalin and phospholipase-A2-receptor have been described as antigens responsible for the formation of the subepithelial immune complexes and renal disease occurrence. In addition, the complement system seems to play a key role in basal membrane damage and in the development of proteinuria in membranous nephropathy. This paper focuses on the common molecular changes involved in the development of nephrotic proteinuria.

## 1. Introduction

A variety of primary and systemic kidney diseases, trigger an excessive protein loss in the urine [1]. Diseases that progress to nephrotic syndrome are grouped into three categories: antibody-mediated diseases (e.g., lupus, membranoproliferative glomerulonephritis, and membranous nephropathy); metabolic disorders (e.g., diabetes, amyloidosis, and Fabry disease) and podocytopathies [2, 3]. The main primary glomerulopathies that course with proteinuria are podocytopathies (minimal change disease and focal segmental glomerulosclerosis), and membranous nephropathy. The podocytopathies are characterized by changes in podocytes. These changes may be at the structural or molecular levels, and some proteins have been shown as a pivot of renal injury and proteinuria development [4].

## 2. Podocytopathies

Podocyte dysfunction may have idiopathic, genetic, or reactive etiologies. The latter involves response to various insults, including mechanical stress, medications, toxins, viral infections, and as yet unidentified circulating proteins [5]. focal adhesion kinase (FAK) plays an important role in the foot process effacement commonly seen in podocytopathies [6, 7]. 

Focal segmental glomerulosclerosis (FSGS) involves changes in the slit membrane [8, 9] as well as in the cytoskeleton and cell stress [8–10]. Furthermore, FSGS may occur due to a collapse of the capillary loop, which could be HIV-1 associated [11]. Mutations in *WT1* gene can also cause Denys-Drash syndrome [12]. Alterations in podocyte proteins or mutations in their coding gene play an important role in the pathogenesis of podocytopathies. Changes in nephrin, as well as in the nephrin homologue Neph1 [13], in CD2-associated protein (CD2AP) [14, 15], in mFAt1 [16], or in podocin [17] have been described. Autosomal recessive changes in *NPHS2* gene causes steroid-resistant nephrotic syndrome [12, 18, 19]. *NPHS2* gene polymorphisms cause proteinuria in patients with minimal change disease (MCD) [20]. It has been proposed that apoptosis, necrosis, or loss of cellular adhesive interaction induce podocyte detachment from the GBM, playing a central role in the FSGS-mediated hyperfiltration process. Modifications in the foot process cytoskeleton may lead to nephrotic syndrome development, being podocalyxin believed to be responsible for foot process stability [21, 22]. Another component of the luminal membrane is a transmembrane tyrosine-phosphatase named GLEPP-1, which might function as a receptor [23] or regulate both the pressure and the filtration rates [24]. The expression of GLEPP-1 seems to be downregulated in patients with FSGS and collapsing glomerulopathy but at normal levels in MCD cases [25]. It has been recently demonstrated that podocalyxin is increased in nephrotic syndromes [26]. 

The pathogenesis of glomerular sclerosis seen in FSGS might to be caused by an increase in glomerular profibrotic cytokines, such as IL-13 and IL-4 [27–30], whereas other studies suggested the increase of TGF-*β* levels in this process [30, 31].

The TGF-*β* pathway controls cellular responses to many chronic glomerular injuries, thereby leading to an increase in the production of extracellular matrix, an increase in podocyte number and area, and apoptosis [32–37]. The TGF-*β* signaling pathway may act as a key mediator in cellular mechanisms responsible for glomerulosclerosis and interstitial fibrosis [35]. A mediator of TGF-*β* signaling, Smad7, is strongly expressed in clinical cases of podocyte injury. In vitro culture of mice podocytes in the presence of TGF-*β* showed that both Smad7 and TGF-SS1 are related to cell apoptosis, suggesting that Smad7 participates in the progressive reduction of podocytes [38]. 

Some studies have shown that higher renal expression of TGF-*β*1 would be observed in children with FSGS in comparison to patients with MCD. This finding suggests that TGF-*β*1 gene transcription in the kidneys may suggest the development of FSGS renal lesions [39].

Evidences suggested that podocytes may succumb the epithelial-mesenchymal transition (EMT) after antigenic encounter. In this phenomenon, podocytes lack their specific epithelial cell markers such as nephrin, P-cadherin, and zonula occludens-1 and acquire markers specific for mesenchymal cells such as desmin, fibroblast-specific protein-1, matrix metalloproteinase-9, type I collagen, *α*-smooth muscle actin, and fibronectin. These changes may lead to a damage in glomerular filtration barrier, which results in proteinuria [40–42].

Elevated TGF-*β*1 production might induce the expression of integrin-linked kinase (ILK), a protein that is related to the pathogenesis of many nephropathies that course with proteinuria. The upregulation of ILK in the podocytes may determine the occurrence of EMT in these cells via snail transcription factor induction [43].

Podocytes of patients with FSGS and membranous nephropathy present lower expression of nephrin mRNA than cells from patients with MCD [44]. Apparently, EMT events are more frequently associated to FSGS and membranous nephropathy than to MCD.

Nephrin is an important component of the slit diaphragm. It also functions as a potent recruiter of other proteins to podocyte membrane such as podocin and CD2AP [45]. Intracellular domains of the nephrin protein are tyrosine phosphorylated by Src family kinases [46]. The phosphorylated tyrosine residues of nephrin might bind to Nck adaptor proteins and consequently induce a local polymerization of actin [47, 48]. In adult mice, it has been shown that inhibition of Nck expression in podocytes, promoted a fast induction of proteinuria, glomerulosclerosis, and the morphological changes observed in foot processes. These results suggest that Nck proteins might contribute to keep intact the glomerular filtration barrier in adults [49]. 

The foot processes are composed by actin cytoskeleton which main components are actin itself, *α*-actinin, and synaptopodin [50, 51]. Some nephrotic syndromes may present a cytoskeleton reorganization after upregulation of *α*-actinin [21]. Dominant mutations in *α*-actinin-4 (ACTN4) gene are associated with FSGS occurrence [52]. The expression of synaptopodin is generally preserved in nephrotic syndromes as MCD, but reduced in FSGS [53]. The expression of other podocyte cytoskeleton-bound proteins, such as the dystroglycans, are kept unaltered in FSGS, but decreased in active MCD [54]. Some studies suggest an important interaction between actin cytoskeleton structure and some components of the slit diaphragm as podocin, nephrin and CD2AP [55, 56].

The nephrotic syndromes are known by their changes in podocytes as the effacement of podocyte foot processes, as well as structural changes in cytoskeleton and molecular reorganization of slit diaphragm [57]. The B7-1 molecule (CD80) is a transmembrane protein commonly encountered only in the cell surface of B lymphocytes and antigen presenting cells [58–60]. Reiser et al. have shown that under stress conditions B7-1 might be expressed on podocytes, which may cause reorganization of actin cytoskeleton and modulation of molecules component of the slit diaphragm [61]. It suggests that B7-1 might be directly involved in the pathogenesis of the nephrotic syndrome.

Sometimes the differentiation between FSGS and MCD cases is very difficult, mainly when renal biopsies present inadequate numbers of glomeruli. In such cases, the typical FSGS focal sclerosis is unable to be evidenced [4]. In these cases, the discovery of other markers is urgently needed. Malondialdehyde is a lipid peroxidation marker induced by oxidative stress that may occur in acute or chronic nephropathies [62, 63]. It has been observed that urinary and serum levels of malondialdehyde as well as its glomerular expression were elevated in patients with FSGS when compared to MCD cases [64, 65]. This indicates that tissue expression of oxidative stress markers should be considered as differential diagnostic tools.

## 3. Membranous Nephropathy

Membranous nephropathy (MN) is one of the most common causes of the nephrotic syndromes in adults, corresponding to 20 percent of the cases [66–68].

The main pathogenic mechanism involved in MN is the deposition of immune complexes, in subepithelial regions that leads to a progressive thinning of the glomerular capillary [68, 69]. For more than 50 years [66, 67, 69–71], the Heymann nephritis model, was induced in rats immunized with a crude kidney-cortex preparation. The data collected from this model suggested that subepithelial glomerular depositions occur due to the circulating immune complexes, caused by membrane fractions from rat renal brush border [66–69, 71–74]. Additionally, with the advent of the passive Heymann nephritis model, it was observed that rats treated with antibodies directed against brush-border proteins also had the same subepithelial depositions, suggesting that circulating immune complexes are not necessary for this event [75, 76].

Afterwards, megalin was found to be the rat antigen involved in this process. Megalin is expressed in the basal surface of the podocyte foot process, the same subepithelial space where immune complexes are formed. That was the first evidence that podocytes could be engaged with the formation of immune complexes. After the development of genome sequencing techniques, specific megalin epitopes were discovered and were associated with the formation of immune complexes [66, 69, 71–74]. Concomitant with megalin discovery, a new experimental model for MN was established by rabbit immunization with cationic fetal bovine serum. This procedure led to the observation that exclusively immunized animals were capable of having subepithelial IgG and C3 deposits, while the animals that received anionic or neutral serum had complex deposits in the mesangium. This experimental model of Heymann nephritis has been reproduced in dogs, cats, rabbits, rats, and mice [77]. Likewise, proteinuria was more intense in animals that received the cationic serum, showing that podocytes might not play a key role in the formation of immune complexes [72–74, 78]. Renal cortex analysis of mice that developed MN after immunization with fetal calf serum by cDNA microarrays, showed that 175 genes had altered expression in relation to normal kidneys, and metallothionein-1—Mt-1, cathepsin D—CtsD, and laminin receptor LAMR-1-1, previously associated with injury, inflammation, and cell-matrix interactions, were overexpressed. This increase in expression was confirmed by Western blotting, and CtsD and Mt-1 were expressed predominantly in tubulointerstitial compartment and LAMR-1 in glomeruli, distribution evidenced by immunohistochemistry [74].

Other enzymes such as DPP IV, NEP, or aminopeptidase A were also recognized as target antigens for circulating antibodies in animal models. NEP enzyme is located in the Bowman's capsule and proximal tubule in both human and rabbit kidneys. The DPP IV enzyme, however, is also found in podocytes, indicating that these two enzymatic antigens may participate in the pathogenesis of membranous nephropathy [67, 68].

The first human antigen to be linked with an autoimmune cause of the disease was the phospholipase A2 receptor (PLA2R). This antigen is expressed in podocytes and it is a member of the mannose receptor family acting as a transmembrane receptor for secreted phospholipases. The interaction between PLA2R and its specific antibody might potentiate the activation of the complement cascade, which, in turn, might damage the filtration membrane and induce proteinuria [71, 78]. Recent studies have shown an association between the presence of phospholipase A2 receptor (PLA2R) in 70% of patients with MN [79].

The role played by the complement system seems to be essential for the development of the disease. That would be reasonable given the fact that IgG immunoglobulin binding to complement fraction C1q induces proteinuria [66, 69, 71]. Analysis of the glomerular capillary area in kidney biopsies collected from membranous nephropathies demonstrates the presence of C5b-9 membrane attack complexes of the complement system. Animal models demonstrated that podocyte lesions might be mediated by reactive oxygen species (ROS) produced in response to the glomerular membrane damage and the deposition of the immune complexes. These ROS might have their damaging effect on the matrix proteins enhanced by lipid peroxidation. The C5b-9 complex might damage the podocyte DNA directly or through the induction of ROS production. Podocyte lesion might increase the expression of matrix metalloproteinase-9 (MMP-9) in these cells, which might induce collagen IV degradation and alterations in nephrin expression. Thus, lipid peroxidation, complement system activation, and ROS production would provide future therapeutic targets for membranous nephropathy [66, 68, 69, 71, 72]. Another molecule of the complement system which has recently been related to the membranous nephropathy is C4d [80]. C4d is generated by the classical or lectin complementpathway. This fragment is highly stable and covalently binds to cell surfaces. Patients with MN show deposition of C4d in situ and it is believed that this molecule is involved in the pathogenesis of this disease. However, interestingly, such as this deposit is not seen in cases of minimal lesion disease, this molecule has great potential as a tool in the differential diagnosis between these two entities [80].

Immunoglobulin subclasses IgG1 and IgG4 are regularly found in MN, being identically deposited in the glomeruli, but presenting a conflicting expression in patients with antenatal form of the disease. Evaluation of the production profile of IgG1, IgG4, and anti-NEP antibodies have shown that IgG4 alone is not enough to produce nephropathy. Children born from mothers who produced decreased anti-NEP antibody levels, but sustained IgG4 subclass, did not present any renal alteration. On the other hand, children whose mother produced all classes of antibodies did present renal failure at birth. One suitable explanation would be that the Fc portion of the IgG classes would differentially interact with complement system and induce variable cellular lesions [66–68, 70–72, 78].

## 4. Conclusion 

The mechanisms of proteinuria in primary glomerulopathies are complex and depend on all the components of the glomerular filtration barrier. In primary membranous glomerulopathy, some molecules such as megalin and phospholipase A2 receptor have been considered as being the antigens responsible for subepithelial immune complexes, which change the glomerular permeability. In both FSGS and MCD podocytopathies, the molecular changes observed in proteins from the cytoskeleton, cell transmembrane, and slit diaphragm induce foot process effacement and changes in negative charges, resulting in strong proteinuria. The understanding of the mechanisms involved in each clinical entity is extremely important for the best treatment choice and adequate patient followup.

## References

[1] Tryggvason K, Patrakka J, Wartiovaara J (2006). Hereditary proteinuria syndromes and mechanisms of proteinuria. *The New England Journal of Medicine*.

[2] Schnaper HW, Kopp JB (2006). Why kidneys fail: report from an American Society of Nephrology Advances in Research Conference. *Journal of the American Society of Nephrology*.

[3] Pollak MR (2002). Inherited podocytopathies: FSGS and nephrotic syndrome from a genetic viewpoint. *Journal of the American Society of Nephrology*.

[4] Jennette JC, Olson JL (2007). The nephrotic syndrome. *Pathology of the Kidney*.

[5] Barisoni L, Schnaper HW, Kopp JB (2007). A proposed taxonomy for the podocytopathies: a reassessment of the primary nephrotic diseases. *Clinical Journal of the American Society of Nephrology*.

[6] Shirato I (2002). Podocyte process effacement in vivo. *Microscopy Research and Technique*.

[7] Ma H, Togawa A, Soda K (2010). Inhibition of podocyte FAK protects against proteinuria and foot process effacement. *Journal of the American Society of Nephrology*.

[8] Schumacher V, Schärer K, Wühl E (1998). Spectrum of early onset nephrotic syndrome associated with WT1 missense mutations. *Kidney International*.

[9] Kambham N, Markowitz GS, Valeri AM, Lin J, D’Agati VD (2001). Obesity-related glomerulopathy: an emerging epidemic. *Kidney International*.

[10] Kincaid-Smith P (2004). Hypothesis: obesity and the insulin resistance syndrome play a major role in end-stage renal failure attributed to hypertension and labelled ‘hypertensive nephrosclerosis’. *Journal of Hypertension*.

[11] Barisoni L, Bruggeman LA, Mundel P, D’Agati VD, Klotman PE (2000). HIV-1 induces renal epithelial dedifferentiation in a transgenic model of HIV-associated nephropathy. *Kidney International*.

[12] Bariéty J, Nochy D, Mandet C, Jacquot C, Glotz D, Meyrier A (1998). Podocytes undergo phenotypic changes and express macrophagic-associated markers in idiopathic collapsing glomerulopathy. *Kidney International*.

[13] Donoviel DB, Freed DD, Vogel H (2001). Proteinuria and perinatal lethality in mice lacking NEPH1, a novel protein with homology to NEPHRIN. *Molecular and Cellular Biology*.

[14] Shih NY, Li J, Karpitskii V (1999). Congenital nephrotic syndrome in mice lacking CD2-associated protein. *Science*.

[15] Shaw AS, Miner JH (2001). CD2-associated protein and the kidney. *Current Opinion in Nephrology and Hypertension*.

[16] Ciani L, Patel A, Allen ND, Ffrench-Constant C (2003). Mice lacking the giant protocadherin mFAT1 exhibit renal slit junction abnormalities and a partially penetrant cyclopia and anophthalmia phenotype. *Molecular and Cellular Biology*.

[17] Boute N, Gribouval O, Roselli S (2000). NPHS2, encoding the glomerular protein podocin, is mutated in autosomal recessive steroid-resistant nephrotic syndrome. *Nature Genetics*.

[18] Tsukaguchi H, Sudhakar A, Le TC (2002). NPHS2 mutations in late-onset focal segmental glomerulosclerosis: R229Q is a common disease-associated allele. *Journal of Clinical Investigation*.

[19] Koziell A, Grech V, Hussain S (2002). Genotype/phenotype correlations of NPHS1 and NPHS2 mutations in nephrotic syndrome advocate a functional inter-relationship in glomerular filtration. *Human Molecular Genetics*.

[20] Zhu L, Yu L, Wang CD (2009). Genetic effect of the NPHS2 gene variants on proteinuria in minimal change disease and immunoglobulin A nephropathy. *Nephrology*.

[21] Smoyer WE, Mundel P, Gupta A, Welsh MJ (1997). Podocyte *α*-actinin induction precedes foot process effacement in experimental nephrotic syndrome. *American Journal of Physiology*.

[22] Kerjaschki D, Sharkey DJ, Farquhar MG (1984). Identification and characterization of podocalyxin—the major sialoprotein of the renal glomerular epithelial cell. *Journal of Cell Biology*.

[23] Thomas PE, Wharram BL, Goyal M, Wiggins JE, Holzman LB, Wiggins RC (1994). GLEPP1, a renal glomerular epithelial cell (podocyte) membrane protein- tyrosine phosphatase. Identification, molecular cloning, and characterization in rabbit. *The Journal of Biological Chemistry*.

[24] Wharram BL, Goyal M, Gillespie PJ (2000). Altered podocyte structure in GLEPP1 (Ptpro)-deficient mice associated with hypertension and low glomerular filtration rate. *Journal of Clinical Investigation*.

[25] Barisoni L, Kriz W, Mundel P, D’Agati V (1999). The dysregulated podocyte phenotype: a novel concept in the pathogenesis of collapsing idiopathic focal segmental glomerulosclerosis and HIV-associated nephropathy. *Journal of the American Society of Nephrology*.

[26] Kavoura E, Gakiopoulou H, Paraskevakou H (2011). Immunohistochemical evaluation of podocalyxin expression in glomerulopathies associated with nephrotic syndrome. *Human Pathology*.

[27] Yap HK, Cheung W, Murugasu B, Sim SK, Seah CC, Jordan SC (1999). Th1 and Th2 cytokine mRNA profiles in childhood nephrotic syndrome: evidence for increased IL-13 mRNA expression in relapse. *Journal of the American Society of Nephrology*.

[28] Le Berre L, Hervé C, Buzelen F, Usal C, Soulillou JP, Dantal J (2005). Renal macrophage activation and Th2 polarization precedes the development of nephrotic syndrome in Buffalo/Mna rats. *Kidney International*.

[29] Lai KW, Wei CL, Tan LK (2007). Overexpression of interleukin-13 induces minimal-change-like nephropathy in rats. *Journal of the American Society of Nephrology*.

[30] Pereira RL, Reis VO, Semedo P (2012). Invariant natural killer T cell agonist modulates experimental focal and segmental glomerulosclerosis. *PLoS ONE*.

[31] Mesnard L, Keller ADC, Michel ML (2009). Invariant natural killer T cells and TGF-*β* attenuate anti-GBM glomerulonephritis. *Journal of the American Society of Nephrology*.

[32] Border WA, Ruoslahti E (1990). Transforming growth factor-*β*1 induces extracellular matrix formation in glomerulonephritis. *Cell Differentiation and Development*.

[33] Shankland SJ, Hugo C, Coats SR (1996). Changes in cell-cycle protein expression during experimental mesangial proliferative glomerulonephritis. *Kidney International*.

[34] Shankland SJ (1997). Cell-cycle control and renal disease. *Kidney International*.

[35] Schiffer M, Von Gersdorff G, Bitzer M, Susztak K, Bottinger EP (2000). Smad proteins and transforming growth factor-*β* signaling. *Kidney International, Supplement*.

[36] Schnaper HW, Hayashida T, Poncelet AC (2002). It’s a Smad world: regulation of TGF-*β* signaling in the kidney. *Journal of the American Society of Nephrology*.

[37] Border WA, Noble NA (1994). Transforming growth factor *β* in tissue fibrosis. *The New England Journal of Medicine*.

[38] Schiffer M, Bitzer M, Roberts ISD (2001). Apoptosis in podocytes induced by TGF-*β* and Smad7. *Journal of Clinical Investigation*.

[39] Strehlau J, Schachter AD, Pavlakis M, Singh A, Tejani A, Strom TB (2002). Activated intrarenal transcription of CTL-effectors and TGF-*β*1 in children with focal segmental glomerulosclerosis. *Kidney International*.

[40] Li Y, Kang YS, Dai C, Kiss LP, Wen X, Liu Y (2008). Epithelial-to-mesenchymal transition is a potential pathway leading to podocyte dysfunction and proteinuria. *American Journal of Pathology*.

[41] Yamaguchi Y, Iwano M, Suzuki D (2009). Epithelial-mesenchymal transition as a potential explanation for podocyte depletion in diabetic nephropathy. *American Journal of Kidney Diseases*.

[42] Reidy K, Susztak K (2009). Epithelial-mesenchymal transition and podocyte loss in diabetic kidney disease. *American Journal of Kidney Diseases*.

[43] Kang YS, Li Y, Dai C, Kiss LP, Wu C, Liu Y (2010). Inhibition of integrin-linked kinase blocks podocyte epithelial-mesenchymal transition and ameliorates proteinuria. *Kidney International*.

[44] Kim HJ, Lee HH, Yoo HD, Hwa Lee J, Hong ST (2002). Development of a solid-phase binding assay and identification of nonpeptide ligands for the FynB Src homology 2 domain. *Journal of Pharmaceutical and Biomedical Analysis*.

[45] Patrakka J, Tryggvason K (2007). Nephrin—a unique structural and signaling protein of the kidney filter. *Trends in Molecular Medicine*.

[46] Lahdenperä J, Kilpeläinen P, Liu XL (2003). Clustering-induced tyrosine phosphorylation of nephrin by Src family kinases. *Kidney International*.

[47] Li H, Zhu J, Aoudjit L (2006). Rat nephrin modulates cell morphology via the adaptor protein Nck. *Biochemical and Biophysical Research Communications*.

[48] Blasutig IM, New LA, Thanabalasuriar A (2008). Phosphorylated YDXV motifs and Nck SH2/SH3 adaptors act cooperatively to induce actin reorganization. *Molecular and Cellular Biology*.

[49] Jones N, New LA, Fortino MA (2009). Nck proteins maintain the adult glomerular filtration barrier. *Journal of the American Society of Nephrology*.

[50] Andrews PM (1981). Investigations of cytoplasmic contractile and cytoskeletal elements in the kidney glomerulus. *Kidney International*.

[51] Mundel P, Heid HW, Mundel TM, Krüger M, Reiser J, Kriz W (1997). Synaptopodin: an actin-associated protein in telencephalic dendrites and renal podocytes. *Journal of Cell Biology*.

[52] Kaplan JM, Kim SH, North KN (2000). Mutations in ACTN4, encoding *α*-actinin-4, cause familial focal segmental glomerulosclerosis. *Nature Genetics*.

[53] Srivastava T, Garola RE, Whiting JM, Alon US (2001). Synaptopodin expression in idiopathic nephrotic syndrome of childhood. *Kidney International*.

[54] Barisoni L, Mundel P (2003). Podocyte biology and the emerging understanding of podocyte diseases. *American Journal of Nephrology*.

[55] Lehtonen S, Zhao F, Lehtonen E (2002). CD2-associated protein directly interacts with the actin cytoskeleton. *American Journal of Physiology*.

[56] Yuan H, Takeuchi E, Salant DJ (2002). Podocyte slit-diaphragm protein nephrin is linked to the actin cytoskeleton. *American Journal of Physiology*.

[57] Somlo S, Mundel P (2000). Getting a foothold in nephrotic syndrome. *Nature Genetics*.

[58] Henry J, Miller MM, Pontarotti P (1999). Structure and evolution of the extended B7 family. *Immunology Today*.

[59] Henry CBS, Duling BR (1999). Permeation of the luminal capillary glycocalyx is determined by hyaluronan. *American Journal of Physiology*.

[60] Wakem P, Burns RP, Ramirez F (2000). Allergens and irritants transcriptionally upregulate CD80 gene expression in human keratinocytes. *Journal of Investigative Dermatology*.

[61] Reiser J, von Gersdorff G, Loos M (2004). Induction of B7-1 in podocytes is associated with nephrotic syndrome. *Journal of Clinical Investigation*.

[62] Agarwal R (2004). Chronic kidney disease is associated with oxidative stress independent of hypertension. *Clinical Nephrology*.

[63] Ong-Ajyooth L, Ong-Ajyooth S, Parichatikanond P (2006). The effect of *α*-tocopherol on the oxidative stress and antioxidants in idiopathic IgA nephropathy. *Journal of the Medical Association of Thailand*.

[64] Kuo HT, Kuo MC, Chiu YW, Chang JM, Guh JY, Chen HC (2005). Increased glomerular and extracellular malondialdehyde levels in patients and rats with focal segmental glomerulosclerosis. *European Journal of Clinical Investigation*.

[65] Nezhad ST, Momeni B, Basiratnia M (2010). Glomerular malondialdehyde levels in patients with focal and segmental glomerulosclerosis and minimal change disease. *Saudi Journal of Kidney Diseases and Transplantation*.

[66] Ronco P, Debiec H (2005). Molecular pathomechanisms of membranous nephropathy: from heymann nephritis to alloimmunization. *Journal of the American Society of Nephrology*.

[67] Rees A, Kain R (2009). A watershed in the understanding of membranous nephropathy. *Nature Reviews Nephrology*.

[68] Ronco P, Debiec H (2010). Membranous glomerulopathy: the evolving story. *Current Opinion in Nephrology and Hypertension*.

[69] Nangaku M, Shankland SJ, Couser WG (2005). Cellular response to injury in membranous nephropathy. *Journal of the American Society of Nephrology*.

[70] Ronco P, Debiec H (2007). Target antigens and nephritogenic antibodies in membranous nephropathy: of rats and men. *Seminars in Immunopathology*.

[71] Glassock RJ (2010). The pathogenesis of idiopathic membranous nephropathy: a 50-year odyssey. *American Journal of Kidney Diseases*.

[72] Ronco P, Debiec H (2007). Podocyte antigens and glomerular disease. *Nephron*.

[73] Jefferson JA, Pippin JW, Shankland SJ (2010). Experimental models of membranous nephropathy. *Drug Discovery Today*.

[74] Wu CC, Chen JS, Huang CF (2011). Approaching biomarkers of membranous nephropathy from a murine model to human disease. *Journal of Biomedicine and Biotechnology*.

[75] van Damme BJC, Fleuren GJ, Bakker WW (1978). Experimental glomerulonephritis in the rat induced by antibodies directed against tubular antigens: V. Fixed glomerular antigens in the pathogenesis of heterologous immune complex glomerulonephritis. *Laboratory Investigation*.

[76] Couser WG, Steinmuller DR, Stilmant MM (1978). Experimental glomerulonephritis in the isolated perfused rat kidney. *Journal of Clinical Investigation*.

[77] Chen JS, Chen A, Chang LC (2004). Mouse model of membranous nephropathy induced by cationic bovine serum albumin: antigen dose-response relations and strain differences. *Nephrology Dialysis Transplantation*.

[78] Ronco P, Debiec H (2011). Advances in membranous nephropathy: success stories of a long journey. *Clinical and Experimental Pharmacology and Physiology*.

[79] Beck LH, Bonegio RGB, Lambeau G (2009). M-type phospholipase A2 receptor as target antigen in idiopathic membranous nephropathy. *The New England Journal of Medicine*.

[80] Espinosa-Hernandez M, Ortega-Salas R, López-Andreu M (2012). C4d as a diagnostic tool in membranous nephropathy. *Nefrologia*.

